# Quantification of myocardial blood flow with ^82^Rb: Validation with ^15^O-water using time-of-flight and point-spread-function modeling

**DOI:** 10.1186/s13550-016-0215-6

**Published:** 2016-08-01

**Authors:** Mary Germino, Jim Ropchan, Tim Mulnix, Kathryn Fontaine, Nabeel Nabulsi, Eric Ackah, Herman Feringa, Albert J. Sinusas, Chi Liu, Richard E. Carson

**Affiliations:** 1 Biomedical Engineering, Yale University, New Haven, CT USA; 2 PET Center, Diagnostic Radiology, School of Medicine, Yale University, New Haven, CT USA; 3 Yale Cardiovascular Research Center, Section of Cardiovascular Medicine, Department of Internal Medicine, Yale University, New Haven, CT USA; 4 PET Center, Yale School of Medicine, PO Box 208048, New Haven, CT 06520-8048 USA

**Keywords:** Myocardial blood flow, Rubidium-82 PET, Image-derived input function, TOF PET

## Abstract

**Background:**

We quantified myocardial blood flow with ^82^Rb PET using parameters of the generalized Renkin-Crone model estimated from ^82^Rb and ^15^O-water images reconstructed with time-of-flight and point spread function modeling. Previous estimates of rubidium extraction have used older-generation scanners without time-of-flight or point spread function modeling. We validated image-derived input functions with continuously collected arterial samples.

**Methods:**

Nine healthy subjects were scanned at rest and under pharmacological stress on the Siemens Biograph mCT with ^82^Rb and ^15^O-water PET, undergoing arterial blood sampling with each scan. Image-derived input functions were estimated from the left ventricle cavity and corrected with tracer-specific population-based scale factors determined from arterial data. Kinetic parametric images were generated from the dynamic PET images by fitting the one-tissue compartment model to each voxel’s time activity curve. Mean myocardial blood flow was determined from each subject’s ^15^O-water *k*_2_ images. The parameters of the generalized Renkin-Crone model were estimated from these water-based flows and mean myocardial ^82^Rb *K*_1_ estimates.

**Results:**

Image-derived input functions showed improved agreement with arterial measurements after a scale correction. The Renkin-Crone model fit (*a* = 0.77, *b* = 0.39) was similar to those previously published, though *b* was lower.

**Conclusions:**

We have presented parameter estimates for the generalized Renkin-Crone model of extraction for ^82^Rb PET using human ^82^Rb and ^15^O-water PET from high-resolution images using a state-of-the-art time-of-flight-capable scanner. These results provide a state-of-the-art methodology for myocardial blood flow measurement with ^82^Rb PET.

**Electronic supplementary material:**

The online version of this article (doi:10.1186/s13550-016-0215-6) contains supplementary material, which is available to authorized users.

## Background

Cardiac perfusion PET with ^82^Rb is clinically useful for diagnosing coronary artery disease [1–4]. Quantification of myocardial blood flow (MBF) and coronary flow reserve (CFR) can be obtained from ^82^Rb PET but relies on accurately modeling the extraction fraction of rubidium by myocardial tissue, which is nonlinearly related to flow as described by the generalized Renkin-Crone model [5–8]. Uncertainty in the extraction model parameters causes much of the uncertainty in MBF [9].

Several groups have reported generalized Renkin-Crone model parameters for rubidium using canine or human MBF data from microspheres [10], ^13^N-ammonia [5, 11], and ^15^O-water [12, 13]. Most of these studies used older-generation PET systems with 2D or reduced-dose 3D acquisitions. To our knowledge, no extraction fraction estimations have been made using scanners with time-of-flight (TOF) capabilities; for ^82^Rb PET, such systems provide better signal-to-noise ratios than non-TOF systems [14, 15] and parametric images with lower standard error. Further, when point spread function (PSF) modeling is included in reconstruction, MBF estimates from ^82^Rb PET may be higher [16]; however, such calculations were performed using an extraction model [11] derived from non-TOF, non-PSF images. Presotto et al. [17] demonstrated the quantitative superiority of PSF + TOF for dynamic cardiac reconstructions using a thorax/heart phantom filled with either ^18^F alone or ^18^F and ^13^N (to simulate dynamically varying contrast), in both static and moving configurations.

Errors in the input function, another critical component of kinetic modeling, can substantially bias kinetic parameter estimates [18]. For practical reasons, image-derived input functions (IDIFs) are widely used in cardiac PET. IDIFs estimated from blood pool regions of cardiac images have been validated against the gold standard of arterial samples in dogs [5, 19] but not recently in humans with ^82^Rb PET.

A recent study of five extraction model fits and three IDIF estimation methods demonstrated that these choices substantially influence MBF estimates [20]. In this work, we reexamined extraction fraction estimation in humans with paired rest and stress studies with ^82^Rb and ^15^O-water acquired on a state-of-the-art system, the Siemens Biograph mCT, and reconstructed images with TOF and PSF modeling. We augmented this high-quality image data with continuously sampled arterial measurements for input function validation. From these data, we provided new parameter estimates of the generalized Renkin-Crone model for rubidium extraction.

## Methods

### Subjects

Nine healthy subjects (five male) with no known cardiac abnormalities were studied. This study was approved by the Yale University Human Investigation Committee; all subjects signed an informed consent form. The average age was 28.4 ± 6.2 years, and average BMI was 24.7 ± 3.9 kg/m^2^. Subjects abstained from caffeine for 12 h pre-imaging, and from food for 4–6 h. Before scanning, an intravenous line for tracer administration and an arterial line for blood sampling were placed.

### Data acquisition

PET scans were acquired on the Biograph mCT 1104 (Siemens Healthcare, Knoxville, TN) at rest and under pharmacological stress with ^82^Rb and ^15^O-water for each subject. For seven subjects, the scan sequence was: ^82^Rb rest; ^15^O-water rest; ^15^O-water stress; and ^82^Rb stress. For the remaining subjects, the ^82^Rb stress acquisition was performed directly after the ^82^Rb rest acquisition. A 1-h interval separated each stress scan from the following acquisition, with confirmation that heart rate and blood pressure had returned to baseline. Low-dose CT attenuation correction scans were acquired before each rest scan and after each stress scan. Pharmacological stress was induced with 0.4 mg of regadenoson, injected over 30 s, 1 min before tracer injection. The ^82^Rb injections were performed with the CardioGen-82 (Bracco Diagnostics, Princeton, NJ) system with an infusion rate of 50 mL/min, duration of 18 ± 4 s, and mean ± SD dose of 663 ± 82 MBq. ^15^O-water infusions with mean dose 690 ± 136 MBq were delivered over 20 s. Dosing was independent of body weight.

### Arterial blood sampling and data analysis

Arterial blood was drawn from the radial artery for 7 min per scan at 4 mL per minute for seven of nine subjects and radioactivity measured with a cross-calibrated radioactivity monitor (PBS-101, Veenstra Instruments, Joure, The Netherlands). One subject chose not to have the arterial line. In another subject, the arterial line was not successfully placed. Because IDIFs were corrected with population-based scale factors, these subjects’ image data were not excluded. Because the 1.25-mL infusion line for ^82^Rb was not flushed, residual activity remained at end-of-elution. This unshielded activity contributed to the background signal detected by the radioactivity monitor, visible in the initial portion of the ^82^Rb arterial readings before the input function peak (when measurements should be 0). To model this background signal, a decaying exponential with the ^82^Rb decay constant was fit to the raw count data for each acquisition, between end-of-elution and the rise of the input function peak. This fitted curve was subtracted from the arterial measurements (see Additional file 1, page 1). Apart from removal of the background signal, ^82^Rb and ^15^O-water data were processed analogously. Corrections were applied for sensitivity, decay, and external dispersion. Sensitivity was measured by cross-calibration with phantom measurements per isotope. To correct for time delay between the left ventricle (LV) and the arterial sampling site, each acquisition’s time shift was estimated by maximizing the correlation between the corrected arterial samples and the LV time activity curve (TAC).

### Image reconstruction

For each injection, list-mode data were acquired for 4 min post-injection and reconstructed into 32 frames (20x3s,6x10s,6x20s) with mCT software using TOF and PSF modeling, and, for ^82^Rb, prompt-gamma correction (OSEM with 2 iterations of 21 subsets, voxel size 2.036x2.036x2.0 mm^3^). Images were post-smoothed with a 3 mm-FWHM Gaussian kernel. Summed dynamic PET images were inspected for alignment with the corresponding attenuation correction CT images, and manually realigned and re-reconstructed as necessary. Images were transformed to short-axis orientation.

### Kinetic modeling

The one-tissue compartment model

1 $$ {C}_{\mathrm{T}}(t)={K}_1{e}^{-{k}_2t}\otimes {C}_{\mathrm{A}}(t) $$

was used to describe the kinetics in the myocardium tissue, where *K*_1_ is the myocardial influx rate constant, *k*_2_ is the efflux rate constant, and *C*_T_(*t*) is the tissue TAC [11, 21, 22]. Tissue TACs were converted from Bq/mL to Bq/g with an assumed tissue density of 1.05 g/mL. The arterial input function *C*_A_(t) was estimated from either the arterial samples or the images (described below).

Partial volume, motion effects, and arterial blood volume were accounted for with one or two additional parameters: *V*_A_, for the LV cavity spillover and arterial blood volume, and, optionally, *V*_RV_, for the right ventricle (RV) cavity spillover:

2 $$ {C}_{\mathrm{PET},\mathrm{m}\mathrm{y}\mathrm{o}}(t)=\left(1-{V}_{\mathrm{A}}-{V}_{\mathrm{RV}}\right){C}_{\mathrm{T}}(t)+{V}_{\mathrm{A}}{C}_{\mathrm{A}}(t)+{V}_{\mathrm{RV}}{C}_{\mathrm{RV}}(t) $$

**Fig. 1:**
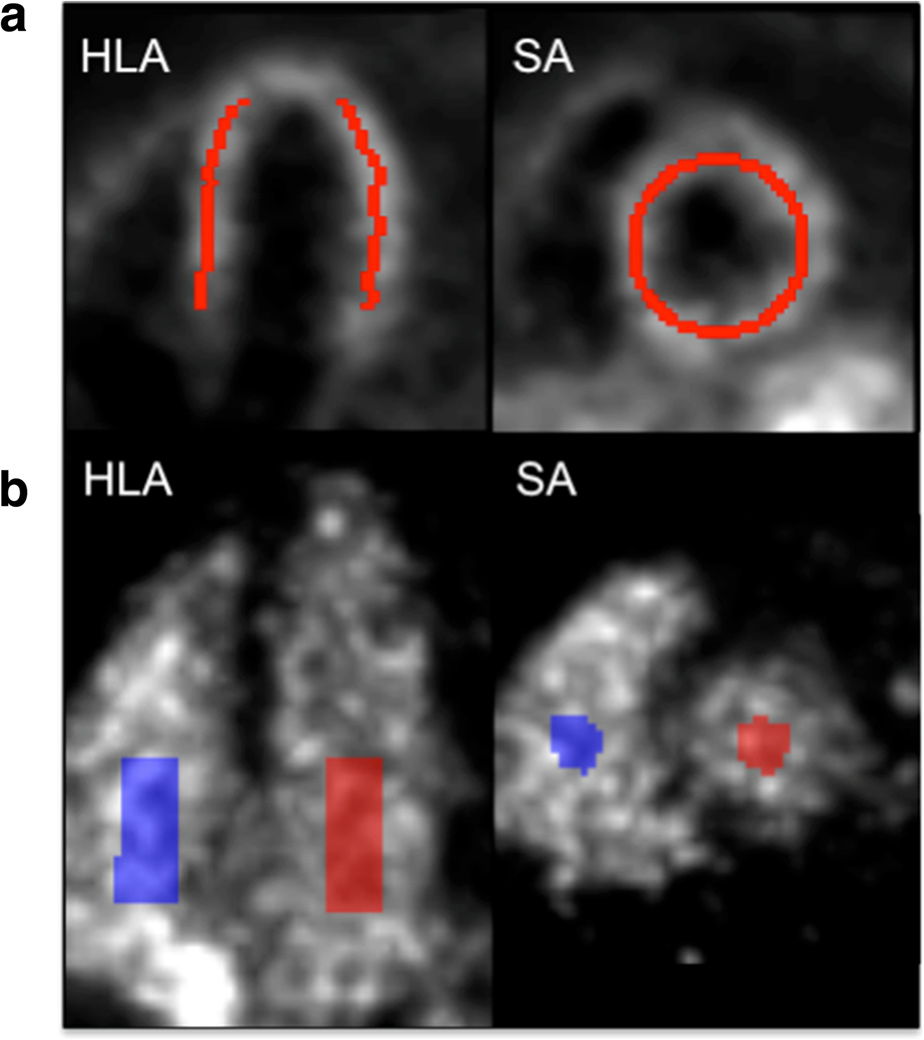
**a** Example myocardium volume of interest (VOI), overlaid on a *K*_1_ image in horizontal long axis (HLA) and short axis (SA). **b** Example VOI for estimating image-derived right ventricle (*blue*) and left ventricle (*red*) TACs, overlaid on a composite image of three early ^82^Rb frames

Equation 2 was fit to each voxel TAC using the basis function method [23] and a weighted least squares (WLS) criterion, with weights based on noise equivalent counts. ^15^O-water *K*_1_ images were registered to the corresponding ^82^Rb *K*_1_ images for each subject/condition using rigid 3D versor transforms optimized by the Mattes mutual information metric. The *k*_2_, *V*_A_, and *V*_RV_ images were realigned using the same transforms. Left ventricular myocardium volumes-of-interest (VOIs) with approximate thickness of 4 mm were automatically determined for each subject’s rest and stress scans from *K*_1_ images, using in-house software (Fig. 1a). Model fits were performed both as three-parameter (without *V*_RV_) and four-parameter fits.

### Input function estimation

IDIFs were estimated from fixed-volume (6.5 mL) cylindrical VOIs manually placed towards the base of the LV and atrium blood pools of each image (Fig. 1b). The resulting TACs were compared to the measured arterial input functions (AIFs) with regard to peak concentration, tail concentration, and area under the curve (AUC). For comparisons, AIFs were resampled to the image times by averaging values within each frame. Peaks were computed as the maximal activity of each TAC. Tail activity was computed as the average concentration over 1 min starting at 2 min, 40 s post-injection. Percent difference in each metric was computed for corresponding pairs of IDIFs and AIFs and averaged across subjects.

With a sufficiently small LV VOI, activity is often assumed to be fully recovered [10–12]; alternatively, IDIFs are sometimes corrected for partial-volume effects. For instance, [13] assumed the LV cavity TAC is a partial-volume mixture of 85 % arterial blood and 15 % myocardial tissue. We investigated partial-volume correction (PVC) methods using the AIF as the gold standard.

To assess IDIF PVC, the “true” tissue TAC *C*_T_ was estimated from a mean global myocardium TAC using the AIF in Eq. 1 and 2 (omitting the *V*_RV_ term). First, a one-parameter model was investigated, where the LV recovery coefficient *β* was estimated from the LV TAC *C*_PET,LV_ and AIF:

3 $$ {C}_{\mathrm{PET},\mathrm{L}\mathrm{V}}(t)=\beta {C}_{\mathrm{A}}(t)+\left(1-\beta \right){C}_{\mathrm{T}}(t). $$

An alternative two-parameter model did not constrain the sum of the coefficients to 1:

4 $$ {C}_{\mathrm{PET},\mathrm{L}\mathrm{V}}(t)={\beta}_1{C}_{\mathrm{A}}(t)+{\beta}_2{C}_{\mathrm{T}}(t). $$

The sum *β*1 + *β*2 might be <1 if partial-volume mixing occurs with signal outside the heart (e.g., the lung).

To perform scale correction, the parameter *β*_s_ is estimated from:

5 $$ {C}_{\mathrm{PET},\mathrm{L}\mathrm{V}}(t)={\beta}_{\mathrm{S}}{C}_{\mathrm{A}}(t). $$

This correction was used by [5], with tracer-independent *β*_s_ ≈ 0.90 estimated from canine ^82^Rb and ^13^N-ammonia PET and well counter measurements of arterial samples.

An alternative scale correction *β*_AUC_ was estimated as the ratio of the IDIF AUC to the AIF AUC:

6 $$ {\displaystyle {\int}_0^T}{C}_{\mathrm{PET},\mathrm{L}\mathrm{V}}(t)\mathrm{d}\mathrm{t}={\beta}_{\mathrm{AUC}}{\displaystyle {\int}_0^T}{C}_A(t)\mathrm{d}\mathrm{t} $$

where *T* is the duration of the dynamic acquisition. While individually estimated *β*_AUC_ cannot outperform *β*_s_ in terms of weighted sum-of-squared residuals (WSS) (*β*_s_ minimizes WSS by design), a population-based *β*_AUC_ might give better kinetic parameter concordance.

Parameters for the one-parameter PVC, two-parameter PVC, and scaling models (Eq. 3, 4, and 5, respectively) were estimated via WLS for each acquisition. Model fits were compared by *F* tests. The ^82^Rb *K*_1_ estimates and ^15^O-water *k*_2_ estimates from AIFs were compared to those from *β*_AUC_ scale-corrected IDIFs by linear Deming regression, which models error in both variables, and by the Lin concordance coefficient [24], which provides a measure of absolute agreement between two estimates.

### MBF estimation

Using myocardial VOIs (Fig. 1a), MBF was estimated from the mean myocardial ^15^O-water *k*_2_ values, corrected with a partition coefficient of *p* = 0.91 mL/g (MBF = *k*_2_*p*) [25]. Finally, the parameters *a* and *b* of the generalized Renkin-Crone model [5, 7, 8]

7 $$ {K}_1=\mathrm{M}\mathrm{B}\mathrm{F}\cdot \left(1-a{e}^{-b/\mathrm{M}\mathrm{B}\mathrm{F}}\right) $$

were estimated from the mean myocardial ^82^Rb *K*_1_ values and ^15^O-water MBF. In this model, *b* reflects the basal permeability-surface area (PS) product and *a* accounts for MBF-dependent PS changes. Fits used weighted orthogonal distance regression [26] to account for errors in both variables, with weights set to the reciprocals of the variance of voxel values in the myocardium VOIs. There were two datapoints per subject (rest and stress). Renkin-Crone parameters were independently estimated from mean kinetic parameters using (1) uncorrected IDIFs, (2) scale-corrected IDIFs, and (3) AIFs. Analyses were also performed without the *V*_RV_ term and separately for the lateral and septal walls.

## Results

### Hemodynamics

**Fig. 2:**
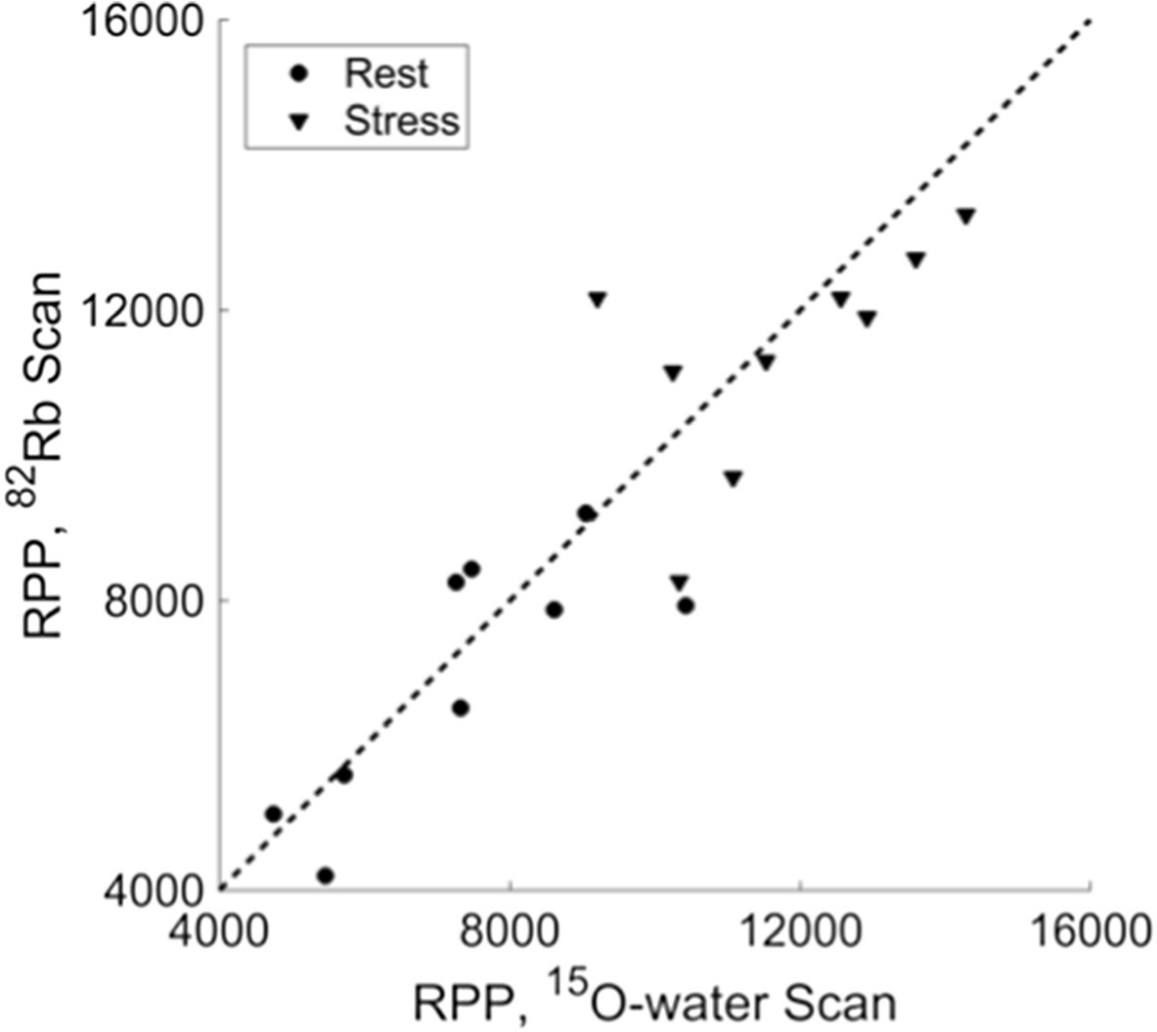
Rate-pressure products (RPP) for ^82^Rb scans versus ^15^O-water scans. The *dashed line* is the identity line. Rest RPP was measured immediately before scan start. Stress RPP is averaged over 4 min post-injection

Figure 2 compares rate-pressure products (RPPs) for each pair of ^82^Rb and ^15^O-water scans. Mean (±standard deviation) absolute percent difference between ^82^Rb and ^15^O-water RPP was 12 ± 9 % at rest and 11 ± 9 % under pharmacological stress. Two subjects had RPPs with greater than 20 % difference between ^82^Rb and ^15^O-water scans at rest, and two subjects had RPPs with greater than 20 % difference between ^82^Rb and ^15^O-water scans under pharmacological stress. No significant group differences between ^82^Rb and ^15^O-water RPPs were found for rest (*p* = 0.40) or stress (*p* = 0.44).

### IDIF validation

Table 1 gives means and standard deviations of the percent differences (across seven subjects) in peak activity, tail activity, and AUC of uncorrected IDIFs with respect to AIFs. The peaks of the ^82^Rb IDIFs were underestimated by 13 and 19 % (rest, stress) compared to the AIFs, with smaller bias in AUC. An ideal IDIF might be expected to have a higher peak value, since no correction for internal-body dispersion was applied to the AIFs. Mean percent differences for all ^15^O-water metrics were within ±10 %. These results suggest that IDIF correction could be beneficial for ^82^Rb kinetic modeling, due to poorer resolution from larger positron range and higher myocardium-to-blood-pool contrast.

**Table 1:** Comparison of uncorrected IDIFs to AIFs

		AUC	Peak	Tail
		% difference mean ± SD	% difference mean ± SD	% difference mean ± SD
^82^Rb	Rest	−11 ± 12	−13 ± 9.2	−7.1 ± 18
	Stress	−6.0 ± 13	−19 ± 18	2.6 ± 23
^15^O-water	Rest	−5.1 ± 10	−8.4 ± 14	−5.4 ± 9.0
	Stress	−1.2 ± 9.9	2.4 ± 18	−3.5 ± 7.8

*SD* standard deviation, *AIF* arterial sample-based input function, *IDIF* image-derived input function, *AUC* area under curve, *% difference* 100 × (IDIF − AIF)/AIF

**Fig. 3:**
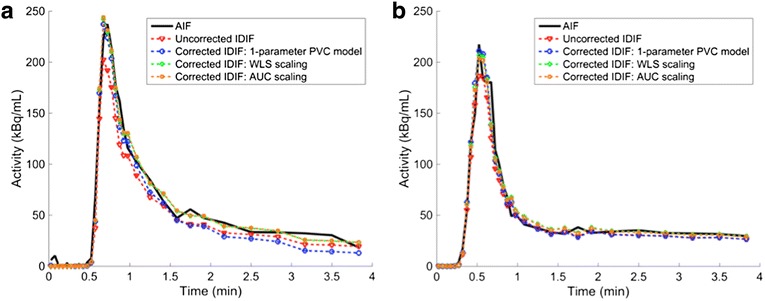
Arterial input functions (AIFs) and image-derived input functions (IDIFs) corrected using parameters estimated for these scans from a typical **a** ^82^Rb scan and **b** ^15^O-water scan. In both cases, weighted-least-squares (WLS)-based scaling (green) and area-under-the-curve (AUC)-based scaling corrections are nearly identical. The two-parameter (partial-volume-corrected) PVC IDIFs were omitted from these plots, as they were virtually identical to the scale-corrected IDIFs (green, orange curves)

The mean and standard deviation of the parameter *β* from the one-parameter PVC model (Eq. 3) was 0.87 ± 0.09 (0.86 ± 0.12) for ^82^Rb (^15^O-water), which are similar to the 0.85 assumed by [13]. However, the tails of ^82^Rb IDIFs corrected by this method were consistently underestimated (−54 ± 29 %) compared to the AIFs; neither the two-parameter PVC model (Eq. 4) nor scaling model (Eq. 5) demonstrated this deficiency (Fig. 3a). For ^15^O-water IDIFs, there was no apparent qualitative difference among correction methods (Fig. 3b).

Using the two-parameter PVC method, the mean and standard deviation of *β*_1_ + *β*_2_ (Eq. 4) were 0.85 ± 0.10 (0.98 ± 0.10) for ^82^Rb (^15^O-water). This indicates that while a partial-volume mixture model of arterial blood and myocardium tissue may be sufficient for water, rubidium could require a different model of recovery-diminishing effects.

The mean and standard deviation of the scaling parameter *β*_s_ (Eq. 5) was 0.83 ± 0.09 (0.94 ± 0.10) for ^82^Rb (^15^O-water). The scaling parameter *β*_AUC_ (Eq. 6) was 0.92 ± 0.12 (0.97 ± 0.10) for ^82^Rb (^15^O-water). Additional file 1: Table S1 gives mean estimated correction parameters by tracer and condition.

Additional file 1: Figure S3 gives results of F-tests comparing the two-parameter PVC model to either the one-parameter PVC or scaling model (Eq. 5) for each acquisition. For 11 of 14 ^82^Rb scans, the two-parameter PVC model outperformed the one-parameter PVC model. For only four ^82^Rb scans, the two-parameter model outperformed scaling with *β*_s_. For most ^15^O-water acquisitions, the two-parameter model was not superior to either one-parameter model (Eqs. 3 and 5).

The AUC scale correction (Eq. 6) cannot be compared to scaling with *β*_s_ by F-test, since the WSS of the AUC scale correction will always be at least that of the *β*_s_ correction. With the *β*_s_ correction, the difference in IDIF peak compared to AIF peak was 1 ± 17 % (3 ± 17 %) for ^82^Rb (^15^O-water); the difference in AUC becomes 10 ± 15 % (3 ± 10 %) for ^82^Rb (^15^O-water). With the *β*_AUC_ correction, the difference in peaks becomes −9 ± 16 % (0 ± 17 %) for ^82^Rb (^15^O-water); the difference in AUC becomes 0 ± 13 % (0 ± 10 %) for ^82^Rb (^15^O-water). Though *β*_s_ correction provides better peak agreement_,_*β*_AUC_ correction improves the peak agreement while also improving AUC agreement.

Based on these results, AUC-based scaling correction was adopted. All IDIFs were corrected by multiplication with the reciprocal of the average *β*_AUC_ per tracer (1.09 for ^82^Rb, 1.03 for ^15^O-water). Eleven of 14 ^82^Rb and 10 of 14 ^15^O-water IDIFs had better agreement (lower WSS) with the AIF after scaling, with an average decrease in WSS of 18 ± 23 and 3 ± 16 %, respectively.

### Parametric images

**Fig. 4:**
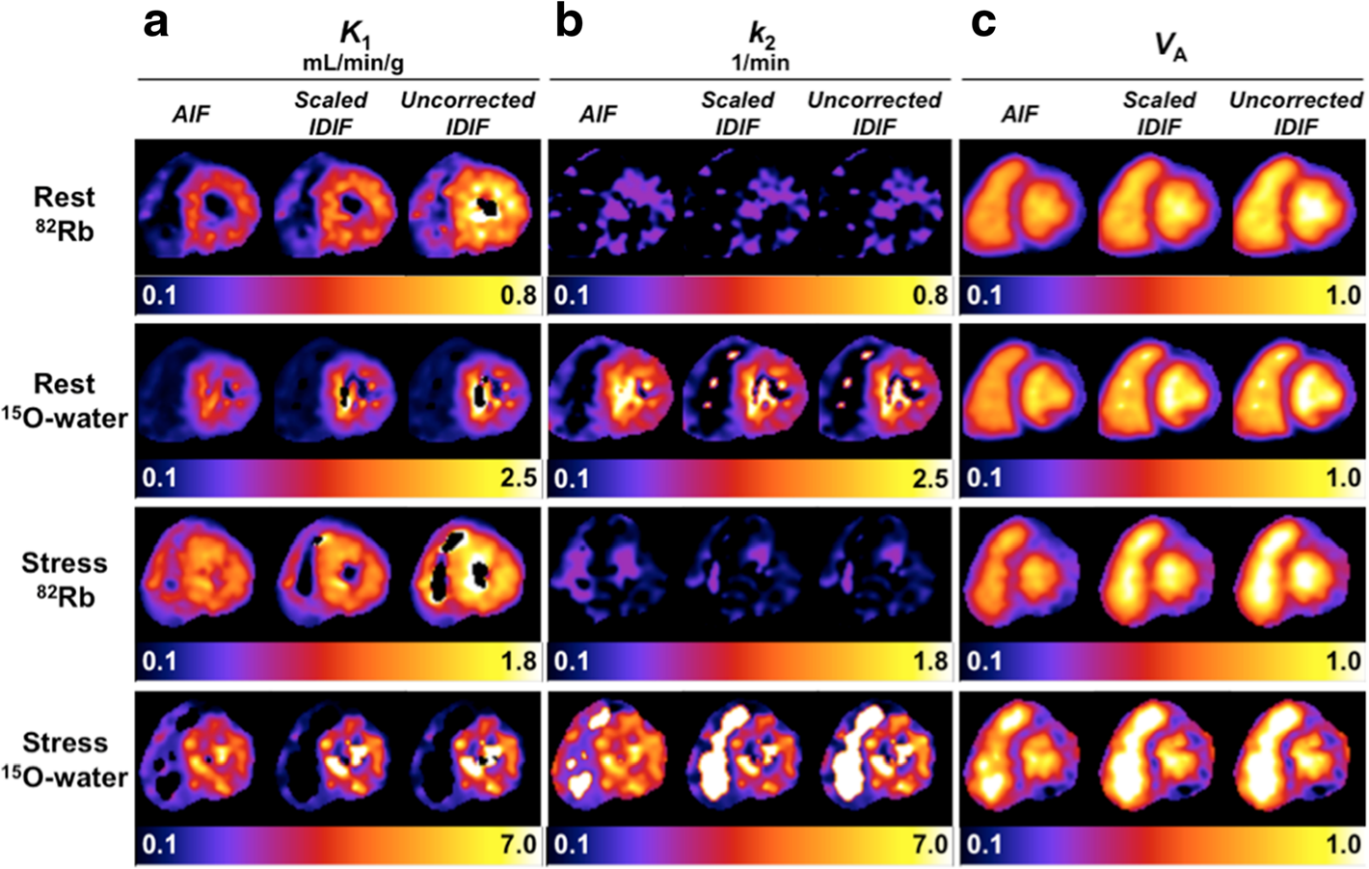
Short-axis parametric images for one subject’s ^15^O-water and ^82^Rb rest and stress scans, generated with different input functions. **a** *K*_1_ parametric images, **b** *k*_2_ parametric images, and **c** *V*_A_ parametric images. Background outside the heart has been omitted for display. *AIF* arterial sample-based input function, *IDIF* image-derived input function. Right ventricle spillover correction term (*V*_RV_) not included

Example parametric images for one subject from the three-parameter fit are shown in Fig. 4, using each of: AIF, scaled IDIF, and uncorrected IDIF (Additional file 1: Figure S4 shows parametric images with and without the *V*_RV_ term). *K*_1_ is an estimate of MBF for ^15^O-water and nonlinearly related to MBF for ^82^Rb due to incomplete extraction, so myocardial *K*_1_ (Fig. 4a) is lower for ^82^Rb than ^15^O-water (note different display scales). Naturally, stress values exceed rest values for both tracers. Because we studied healthy subjects, *K*_1_ is relatively uniform in the myocardium. The LV cavity is more blurred in the *K*_1_ images at stress than at rest for both tracers, presumably due to greater motion during stress. *K*_1_ images are noisiest for ^15^O-water stress, where higher *k*_2_ results in greater correlation between *K*_1_ and *V*_A_, posing a more difficult estimation problem. The noisy ^82^Rb *k*_2_ images (Fig. 4b) show poor delineation of the myocardium due to minimal tracer washout, as rubidium is trapped by viable myocardial tissue. In contrast, ^15^O-water freely diffuses in and out of tissue, so *k*_2_ is proportional to MBF, and the *k*_2_ images mirror the *K*_1_ images. In the blood pool, *k*_2_ values are very noisy since *k*_2_ has minimal effect on model fits with *K*_1_ ≈ 0. Since the *V*_RV_ term was not included here, both the RV and LV blood pools are distinctly visible in the *V*_A_ images (Fig. 4c). *V*_A_ is overestimated from images using the uncorrected IDIF as compared to the AIF or scaled IDIF. The ^15^O-water *V*_A_ images are slightly sharper than the ^82^Rb *V*_A_ images, particularly near the septum, which may be explained by the poorer resolution of ^82^Rb.

For this subject, the ^82^Rb *K*_1_ images from corrected IDIFs showed qualitatively better agreement with those from AIFs than those using uncorrected IDIFs (Fig. 4a). Because corrected IDIFs were generated using a population-based correction factor, not all scans had comparable improvement in agreement between corrected IDIF- and AIF-based images. The IDIF correction factor was closer to unity for ^15^O-water than ^82^Rb, so ^15^O-water *K*_1_ images were less affected by IDIF correction. The IDIF correction had virtually no impact on *k*_2_ parametric images (Fig. 4b), as *K*_1_ and *V*_A_ compensate changes in input function scale.

Table 2 lists mean myocardial parameter estimates across subjects (using subject-specific regions) for the uncorrected IDIFs, scaled IDIFs, and AIFs (three-parameter fit; see Additional file 1: Table S2 for four-parameter results). Standard deviations as a percent of the mean are similar between tracers for *K*_1_. The *V*_A_ estimates, which account for both fractional blood volume and partial-volume effects, are lower for ^15^O-water than ^82^Rb, presumably due to the better resolution of ^15^O. The *K*_1_ data agreed better between the scaled IDIF and AIF estimates than between the uncorrected IDIF and AIF estimates; *k*_2_ estimates were similar for all IFs.

**Table 2:** Mean kinetic parameter estimates from three-parameter fit (without *V*_RV_)

			AIF	Scaled IDIF	Uncorrected IDIF
*K*_1_ (mL/min/g) mean ± SD	Rest	^82^Rb	0.43 ± 0.09	0.45 ± 0.05	0.53 ± 0.06
		H_2_^15^O	0.87 ± 0.21	0.86 ± 0.15	0.91 ± 0.16
	Stress	^82^Rb	0.99 ± 0.19	1.11 ± 0.13	1.30 ± 0.17
		H_2_^15^O	3.43 ± 1.62	3.53 ± 0.85	3.68 ± 0.89
*k*_2_ (1/min) mean ± SD	Rest	^82^Rb	0.11 ± 0.05	0.13 ± 0.04	0.13 ± 0.04
		H_2_^15^O	1.10 ± 0.31	1.05 ± 0.22	1.05 ± 0.22
	Stress	^82^Rb	0.21 ± 0.06	0.23 ± 0.08	0.23 ± 0.08
		H_2_^15^O	3.76 ± 1.24	4.10 ± 1.06	4.10 ± 1.06
*V*_A_ mean ± SD	Rest	^82^Rb	0.32 ± 0.05	0.37 ± 0.04	0.40 ± 0.05
		H_2_^15^O	0.29 ± 0.08	0.33 ± 0.05	0.34 ± 0.06
	Stress	^82^Rb	0.31 ± 0.06	0.40 ± 0.06	0.44 ± 0.06
		H_2_^15^O	0.27 ± 0.06	0.27 ± 0.06	0.28 ± 0.06

*SD* standard deviation, *AIF* arterial sample-based input function, *IDIF* image-derived input function

This study’s mean ^82^Rb rest and stress *K*_1_ and *k*_2_ estimates using scaled IDIFs were comparable to those reported by [11]. The *V*_A_ estimates were approximately 20 % lower than those in [11], likely attributable to IDIF correction and the improved resolution of this study’s images.

**Fig. 5:**
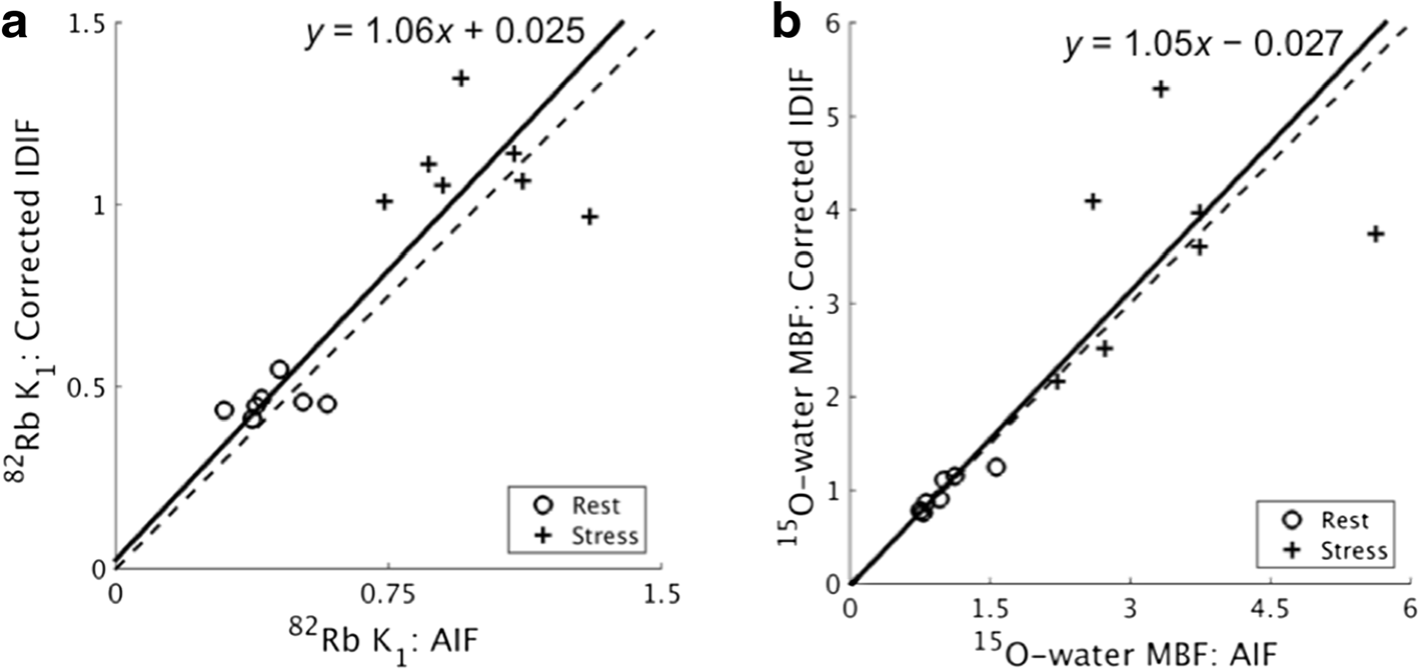
**a** Comparison of mean myocardial ^82^Rb *K*_1_ values using scale-corrected IDIFs vs. AIF. **b** Comparison of mean myocardial ^15^O-water *k*_2_ values. Parameters were estimated with the three-parameter model (omitting right ventricle spillover term). The *solid lines* and *equations* represent fits from Deming regression. The *dashed line* is the identity line. *AIF* arterial sample-based input function, *IDIF* image-derived input function

Figure 5a, b shows the good concordance between corrected IDIF- and AIF-based ^82^Rb *K*_1_ and ^15^O-water *k*_2_ mean myocardial estimates, respectively. Regression slopes (±standard error) for both ^82^Rb *K*_1_ and ^15^O-water *k*_2_ were close to 1.0 (1.06 ± 0.23 and 1.05 ± 0.36, respectively), and both intercepts were nearly 0 (0.025 ± 0.12 and -0.027 ± 0.51, respectively). The concordance correlation coefficient was 0.84 for both ^82^Rb *K*_1_ and ^15^O-water *k*_2_. Datapoints with poorer concordance may indicate subject motion, particularly during stress acquisitions, which affects the accuracy of IDIFs.

### Extraction fraction parameter estimates

**Fig. 6:**
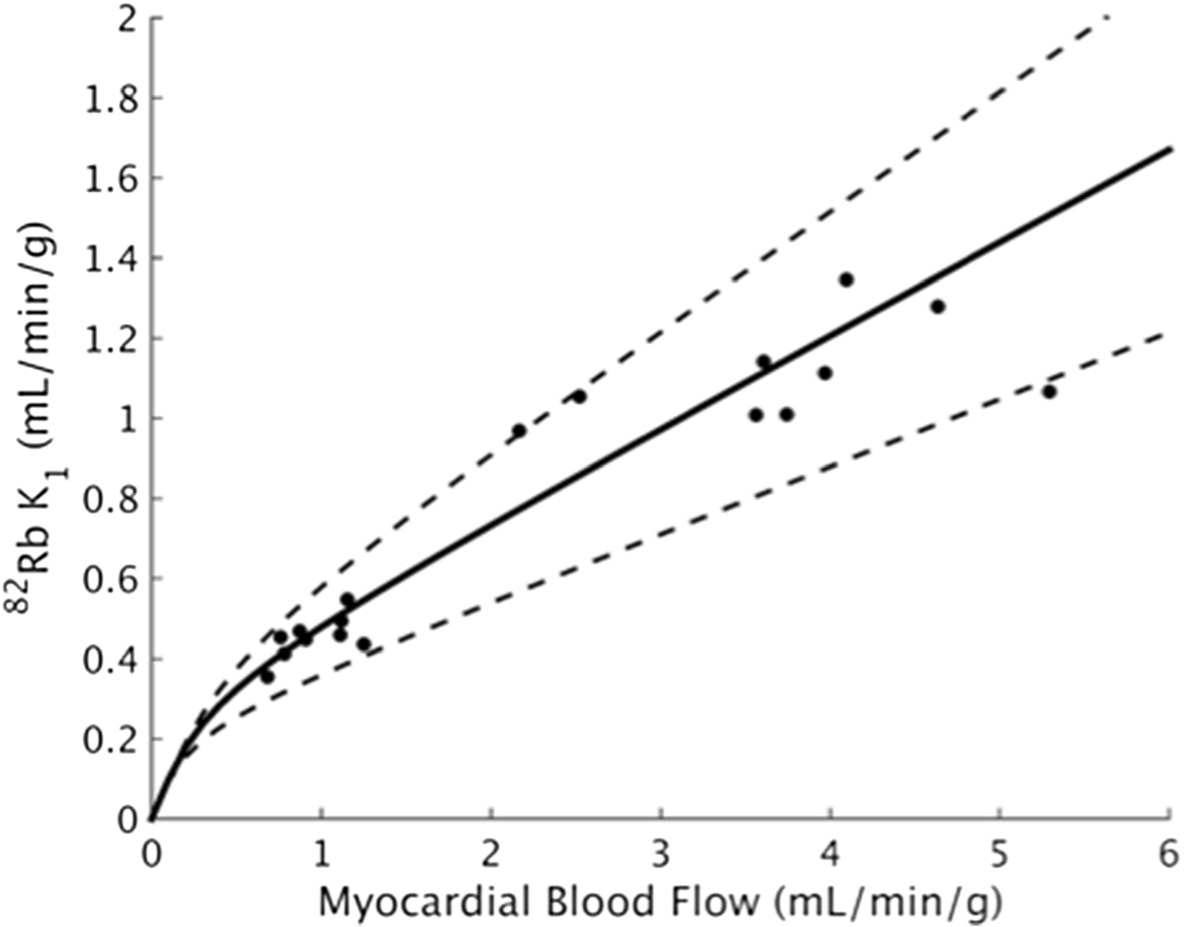
Renkin-Crone model fit of *K*_1_ and myocardial blood flow from three-parameter kinetic model using scale-corrected image-derived input function. The *dashed lines* represent 95 % confidence interval of the regression line

**Fig. 7:**
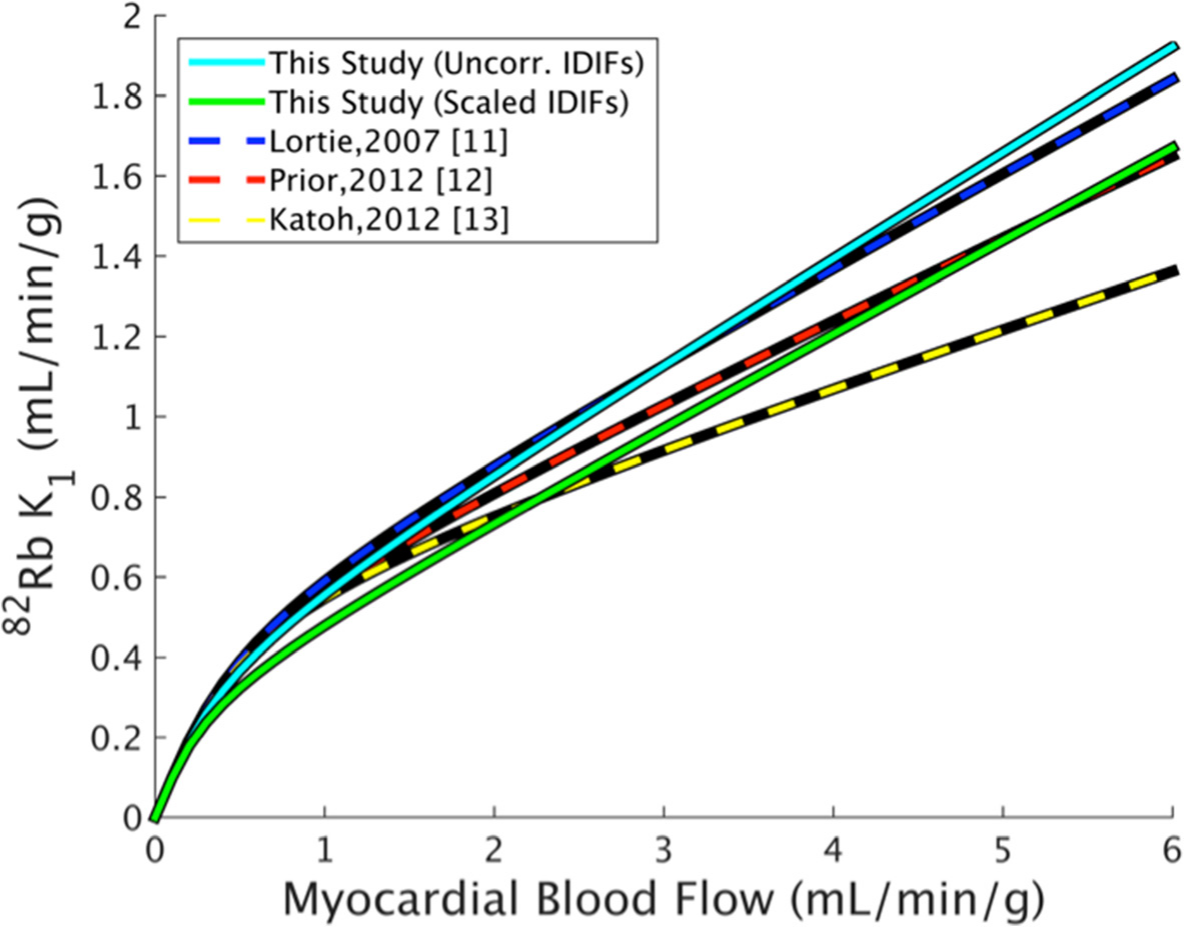
Comparison of this study’s Renkin-Crone model fit to published fits

Figure 6 shows the Renkin-Crone model fits based on three-parameter kinetic model fits using scaled IDIFs; Table 3 gives corresponding parameter estimates. The *V*_RV_ term did not greatly impact Renkin-Crone fits estimated from global myocardial parameters, though omitting it resulted in greater differences between separate septal and lateral regional fits (Additional file 1: Figure S8). Figure 7 and Table 3 compare this study’s extraction model parameter estimates (with and without IDIF correction) to several previously published fits. This study’s *a* estimates are in reasonable agreement with that of the six comparison studies. Though the parameter *b* estimated using uncorrected IDIFs agrees with previous estimates, IDIF correction results in reduced *b*. The standard error of the estimates of *a* and *b* of this study were similar to or lower than those previously published.

**Table 3:** Renkin-Crone parameter estimates from this and published studies

					Renkin-Crone parameter estimates	
	Species	Flow measurement	Kinetic model	Input function correction	*a* ± SE	*b* ± SE
This study	Human	^15^O-water	1TCM	None	0.74 ± 0.03	0.51 ± 0.09
				Scaling	0.77 ± 0.03	0.39 ± 0.06
Yoshida 1996 [5]	Dog	^13^N-ammonia	Retention	Scaling	0.85 ± 0.03	0.45 ± 0.08
Lortie 2007 [11]	Human	^13^N-ammonia	1TCM	None	0.77 ± 0.05	0.63 ± 0.17
Lautamaki 2009 [10]	Dog	Microspheres	1TCM	None	0.89	0.68
Prior 2012 [12]	Human	^15^O-water	1TCM	None	0.80 ± 0.04	0.59 ± 0.14
Katoh 2012 [13]	Human	^15^O-water	1TCM	2-parameter partial-volume correction	0.86	0.54
Renaud 2013 [30]	Human	^13^N-ammonia	Retention	None	0.92	0.74

*IDIF* image-derived input function, *SE* standard error

**Fig. 8:**
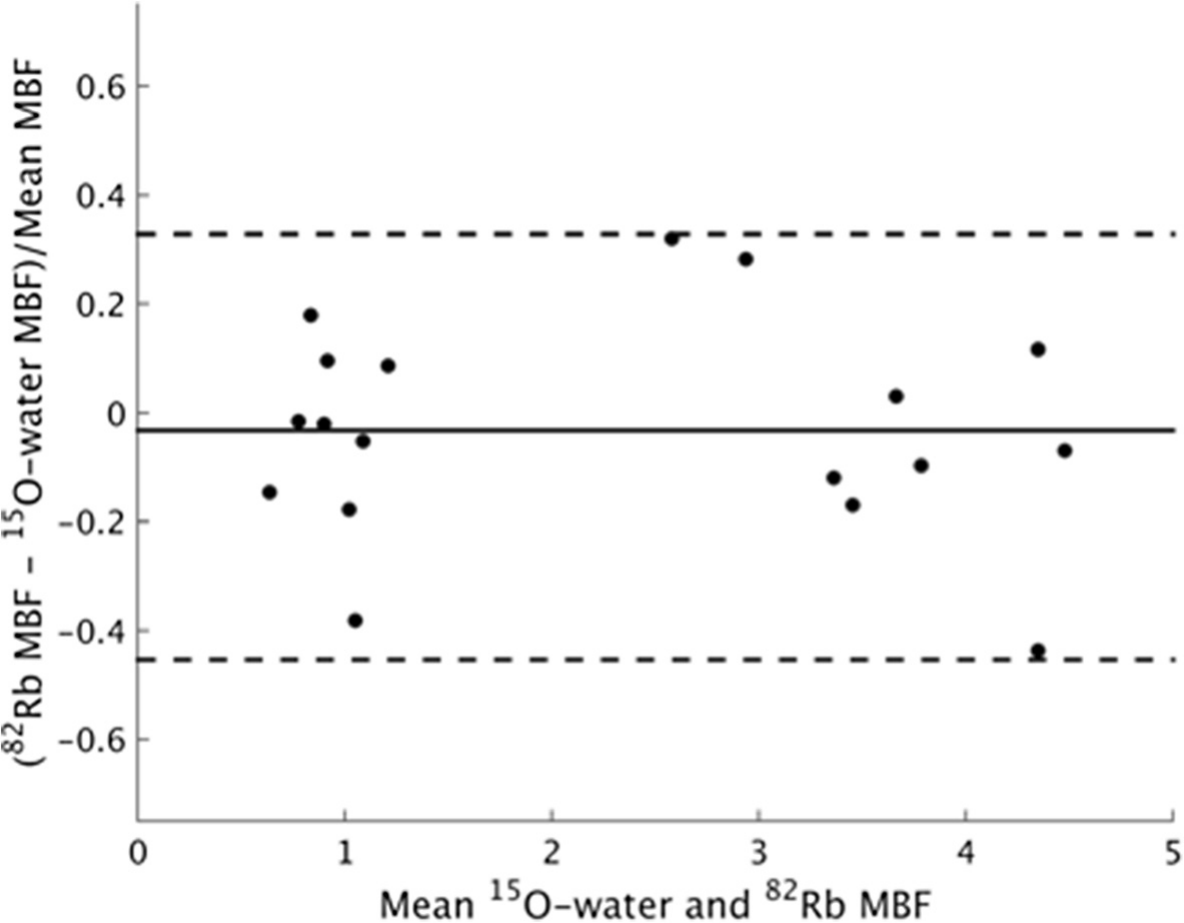
Bland-Altman plot comparing ^15^O-water myocardial blood flow (MBF) to ^82^Rb MBF, using scaled image-derived input functions. Mean percent difference is −3.2 %. The *dashed lines* show 95 % confidence intervals

MBF estimated from ^15^O-water *k*_2_ (^82^Rb *K*_1_) was 0.96 ± 0.20 (0.91 ± 0.19) at rest and 3.73 ± 0.96 (3.59 ± 0.55) under pharmacological stress (Table 4). These flows are consistent with previously published measurements in healthy controls [27]. A Bland-Altman analysis shows no systematic bias between ^82^Rb and ^15^O-water flows (*p* = 0.51 for paired *t* test; Fig. 8). The reproducibility coefficient, defined as 1.96 times the standard deviation of the differences between the water and rubidium-based MBFs, normalized to the mean of each water-rubidium pair, was 39 %. While this study used ^15^O-water MBF estimated from *k*_2_, *K*_1_-based flows were similar (Additional file 1: Figures S7 and S8).

**Table 4:** Extraction-corrected population estimates of myocardial blood flow, mean (±standard deviation)

		^82^Rb MBF (mL/min/g)	^15^O-water MBF (mL/min/g)
Uncorrected IDIF	Rest	0.92 ± 0.19	0.96 ± 0.20
	Stress	3.65 ± 0.64	3.73 ± 0.96
Scaled IDIF	Rest	0.91 ± 0.19	0.96 ± 0.20
	Stress	3.59 ± 0.55	3.73 ± 0.96

*IDIF* image-derived input function, *MBF* myocardial blood flow

## Discussion

This study estimated Renkin-Crone extraction model parameters for rubidium using state-of-the-art ^82^Rb and ^15^O-water TOF PET images with PSF reconstruction. IDIF estimation was validated with continuously sampled arterial blood measurements (AIFs).

Armstrong et al. [16] provided a comparison of reconstructions with TOF and PSF to standard reconstructions; they reported an average increase in MBF of 10–14 % in advanced reconstructions compared to standard OSEM, which is consistent with improved recovery of signal in the myocardium. Because that study used only ^82^Rb, extraction could not be estimated from the advanced reconstructions.

The PSF modeling used here was not isotope-specific. Because ^15^O and ^82^Rb have poorer resolution than ^18^F, the PSF modeling will provide only partial resolution recovery. Though the employed reconstruction should provide better resolution than reconstruction without PSF modeling, there is further room for improvement.

Resolution modeling in PET reconstruction can produce ringing artifacts that significantly impact quantification [28]. These effects are most often detectible in simulation and phantom studies with well-defined object borders. Here, because of the additional blurring incurred by uncompensated cardiac and respiratory motion and modest Gaussian filtering applied pre-modeling, Gibbs-like artifacts were not observed.

While PSF modeling and TOF can provide improved resolution, the primary benefit in this application is reduced noise in the parametric images. For representative maps of the standard error of ^82^Rb *K*_1_ and ^15^O-water *k*_2_, see Additional file 1: Figure S9.

In 3D PET with high injected activities, inaccurate corrections for detector deadtime can impact the accuracy of reconstructed activity in early frames. A previous patient study on the Biograph mCT suggests that doses of ^82^Rb <1110 MBq can avoid significant detector saturation [29]. The doses used in the present study were on average ~60 % of this, and none exceeded it. Based on the peak singles rates, the average peak deadtime was 35 ± 8 % (32 ± 7 %) for ^82^Rb (^15^O-water).

PET image resolution is affected by positron range, detector resolution, smoothing in the reconstruction, and motion. Poor resolution affects quantification of myocardial activity and IDIFs. To minimize the impact of these effects on IDIFs, VOIs are typically small and central in the blood cavity where spill-in and spill-out are presumed insignificant. IDIF accuracy is important to kinetic modeling results; simulations show that a 10 % error in the IF peak can bias ^82^Rb *K*_1_ estimates by up to 25 % [18]. Thus, verification of IDIF accuracy is highly important.

IDIFs had lower correspondence with AIFs for rubidium than water, particularly in terms of peak activity. Unlike water, rubidium is retained in myocardial tissue, causing the tail of the blood pool TAC to fall below that of the myocardium TAC (water IDIFs will have matched activity in the tails of the LV cavity and myocardium TACs). When ^82^Rb images are degraded by motion and resolution effects, expected consequences for IDIFs are reduced peak activity (spill-out from LV cavity) and increased tail activity (spill-in from myocardium). Though prior publications’ images [11, 12] were likely of poorer resolution than this study’s, their IDIFs were uncorrected. In the current study, we primarily observed reduced peak activity in ^82^Rb IDIFs, with differences in tail activity inconsistent with a strict model of geometric PVC (Eq. 3). In Katoh et al. [13], LV TACs were corrected using a PVC model; that model overcorrected the tails of our ^82^Rb IDIFs. A mismatch between ^82^Rb IDIFs and AIFs was better described using a two-parameter model (Eq. 4), which provides for signal mixing with background regions. The simpler scale factor correction (Eq. 5), which recovers activity from an unspecified combination of resolution degradation effects, gave comparable results. For half the ^82^Rb acquisitions, the AUC-based scale factor *β*_AUC_ (Eq. 6) was approximately equal to the scan-specific *β*_s_ (Eq. 5). For the rest, *β*_AUC_ was markedly higher than *β*_s_; in these cases, the IDIF more greatly underestimated the AIF peak, with lower error in the tail. When *β*_s_ is used to correct these cases, though the average peak error is reduced to ~0, the average AUC is overestimated, because tail activity is overestimated.

We chose scaling IDIFs based on AUC matching as more appropriate than WLS-based scaling. For ^15^O-water, the differences between the two methods were small. ^82^Rb, however, is more sensitive to the input function AUC, as its uptake is approximately irreversible. For such tracers, tissue activity is directly proportional to the input function AUC, so errors in the AUC propagate into the parameter estimates.

Given our AIF measurements and anticipating that population-based IDIF correction is most practical for scans without arterial sampling, we used mean tracer-dependent scale factors to correct IDIFs. However, the optimal correction factor is scan-dependent, conditional on variations in VOI size and placement, heart size, breathing pattern, and subject motion. Further investigation is required to assess generalizability to other scanners and reconstruction algorithms. Using AIFs for modeling does not guarantee accurate parameter estimates, as error in myocardial TACs not addressed by the partial-volume fractions *V*_A_ and *V*_RV_ (Eq. 2) could induce bias. Body and respiratory motion are likely principal sources of error. One limitation this study shares with previous publications is that no motion compensation was incorporated.

The Renkin-Crone model fits obtained in this study are similar to previously published fits, though our *b* estimate is lower, primarily a reflection of lower ^82^Rb *K*_1_ estimates in this study compared to others, from the IDIF correction that was applied. With uncorrected IDIFs, extraction parameters obtained in this study closely match those previously published by Lortie [11]. Because scaling correction impacts ^82^Rb *K*_*1*_ but not ^15^O-water *k*_2_, scaled IDIFs resulted in a decrease in the *b* parameter of the Renkin-Crone model. For most previous studies, there was no gold standard measurement of the input function, and therefore, no basis for IDIF correction. Katoh et al. [13] used partial-volume corrected IDIFs, which explains the better agreement between their extraction model and that from corrected IDIFs in this study, compared to the Lortie model. The *a* parameter of the Renkin-Crone model is less sensitive to IDIF correction and reported values vary less across the literature.

Some differences in Renkin-Crone fits can also be explained by the flow estimation method. For instance, [11] used ^13^N-ammonia to measure MBF, which has a lower extraction fraction than ^15^O-water and will hence underestimate MBF. Using unweighted ODR to fit the data instead of weighted ODR resulted in higher parameter estimates (Additional file 1: Figure S10). Average MBFs were similar regardless of IDIF correction because water-based flows were unaffected by IDIF scaling and extraction parameters were estimated separately for each case. Therefore, accurate flows can be determined for ^82^Rb with or without IDIF correction, if extraction parameters have been estimated from data processed similarly. This suggests that modeling choices could have greater impact on extraction fraction estimates than TOF and PSF modeling, though TOF/PSF-based kinetic parameters have lower standard error.

## Conclusions

We have presented parameter estimates for the generalized Renkin-Crone model of extraction for ^82^Rb PET using human ^82^Rb and ^15^O-water PET from high-resolution images from a state-of-the-art TOF-capable scanner with PSF reconstruction. The image-derived input functions were validated against direct arterial measurements, and a scale correction improved the accuracy of IDIFs. With this IDIF correction, MBF should be estimated from ^82^Rb *K*_1_ using the Renkin-Crone parameters reported here. These results provide a state-of-the-art methodology for MBF measurement with ^82^Rb PET, though further validation will be necessary in patients with coronary artery disease with infarcts and ischemia.

## Abbreviations

AIF, arterial input function; IDIF, image-derived input function; LV, left ventricle; MBF, myocardial blood flow; PSF, point spread function; PVC, partial-volume correction; RPP, rate-pressure product; RV, right ventricle; TAC, time activity curve; TOF, time-of-flight; VOI, volume of interest; WLS, weighted least squares

## Additional file

Additional file 1: Figures S1–S10 and Tables S1–S2. (PDF 2.93 mb)
