# Supplementary material for: Quantification of myocardial blood flow with 82Rb: Validation with 15O-water using time-of-flight and point-spread-function modeling
Source: EJNMMI Res. 2016 Aug 1;6:68. doi: 10.1186/s13550-016-0215-6 (PMC5030203; doi:10.1186/s13550-016-0215-6)

Quantification of myocardial blood flow with ^{82}Rb: Validation with ^{15}O-water using time-of-flight and point-spread-function modeling

European Journal of Nuclear Medicine and Molecular Imaging Research

Mary Germino, Jim Ropchan, Tim Mulnix, Kathryn Fontaine, Nabeel Nabulsi, Eric Ackah, Herman Feringa, Albert J. Sinusas, Chi Liu, Richard E. Carson

Corresponding Author: Mary Germino, Yale University, Department of Biomedical Engineering, mary.germino@yale.edu

Correction of ^{82}Rb arterial data to account for background from residual activity in the administration line.

For the ^{15}O-water injections, the administration line from the syringe to the injection site was very short (15 cm), and placed on the opposite side of the subject from the blood radioactivity monitor (PBS-101, Veenstra Instruments, Joure, The Netherlands) such that the subject's body provided some shielding.

For the ^{82}Rb injections, there was a much longer (~110 cm), unshielded administration line from the generator to the subject in direct line-of-sight to the radioactivity monitor. The generator and radioactivity monitor were both placed behind the scanner on opposite sides of the gantry.

Upon examination of the arterial measurements, we noticed that the initial portion of data (before the rise of the input function) was non-zero for the ^{82}Rb scans, but not for the ^{15}O-water scans. Our hypothesis is that the source of the contamination was radioactivity sitting in the patient administration line, because that was the primary difference in the set-up between tracers. For both tracers, the input to the subject injection line was switched to a saline drip post-infusion via a 3-way stopcock (with increased flow for the first minute post-injection). However, this flushed only the subject injection line after the stopcock, and not the longer extent of ^{82}Rb patient infusion line before the stopcock.

To test this hypothesis, we performed a separate experiment (without a human subject) in which we placed the rubidium generator and administration line in the same configuration as used for the human scans. We administered a dose of 740 MBq into a shielded vial placed inside the scanner and recorded the background signal on the counter used for arterial blood measurements, for 5 minutes. The activity present in the administration line produced a measureable background signal that decayed with a halflife of ^{82}Rb (see Online Resource 1 Figure 1).

Therefore, we corrected the subject arterial measurements for this effect by fitting the raw counts measured by the monitor in the beginning of each curve to a ^{82}Rb decay function, and subtracting this background before proceeding with the other corrections (Online Resource 1 Figure 2). Note that we only fit data after the end of elution, as the activity in the administration line is not constant during elution. The magnitude of the background contamination varied with the age of the generator, and correction for this effect resulted in an average reduction in peak activity of 2.8±2.3%. In all cases, this correction brought the arterial input functions into closer agreement with the image-derived input functions.

Fig. S1 Uncorrected measurement of background signal caused by the patient administration line during ^{82}Rb elution into a shielded vial. Red line shows fit to ^{82}Rb decay curve

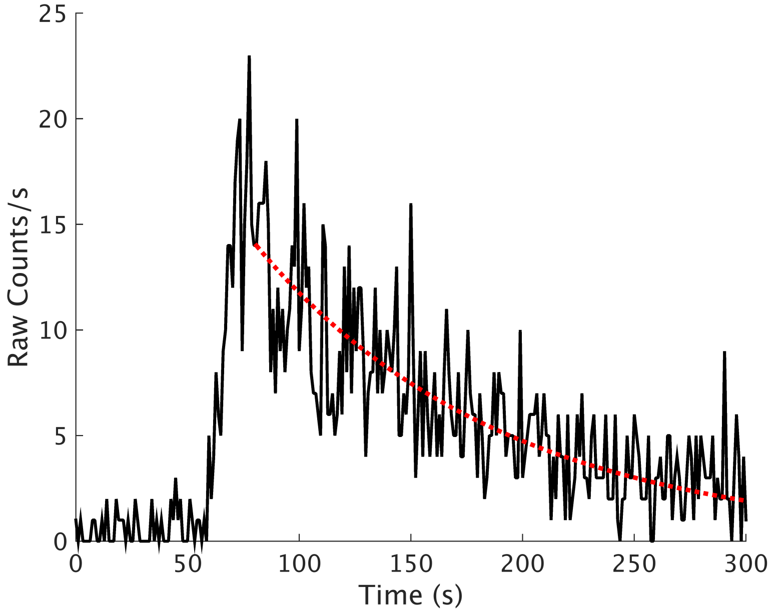

Fig. S2 Uncorrected arterial blood measurements from one scan of a human subject with ^{82}Rb. The cyan curve illustrates the portion of data used to fit of the background signal to a ^{82}Rb decay curve, and the red dashed line illustrates the curve that is subtracted from the raw count data to remove the background signal, prior to other corrections

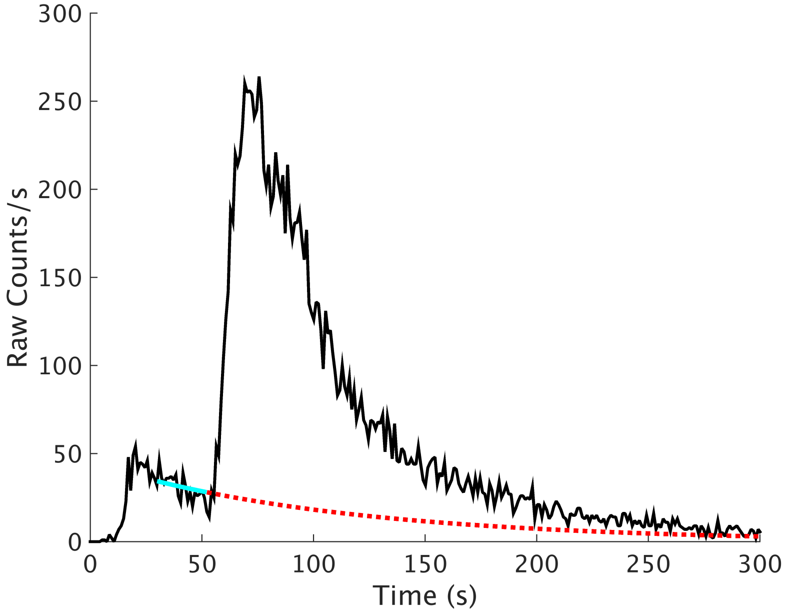

Table S1 Mean estimated IDIF correction parameters by 3 models (n=7).

		1-parameter PVC (Eq. 3): β Mean±SD	2-parameter PVC (Eq. 4): β_1 Mean±SD	2-parameter PVC (Eq. 4): β_2 Mean±SD	Scaling (Eq. 5): β_s Mean±SD	Scaling (Eq. 6): β_{AUC} Mean±SD
^{82}Rb	Rest	0.85±0.07	0.85±0.07	0.002±0.10	0.85±0.09	0.89±0.12
	Stress	0.90±0.10	0.78±0.12	0.066±0.08	0.82±0.10	0.94±0.13
^{15}O-water	Rest	0.88±0.12	0.90±0.13	0.062±0.13	0.92±0.10	0.95±0.10
	Stress	0.85±0.13	0.92±0.23	0.079±0.18	0.97±0.10	0.99±0.10

SD=standard deviation
IDIF=image-derived input function
PVC=partial volume correction

To test the robustness of IDIF correction as a function of LV VOI size, correction factors ($_{AUC}$ in Eq. 6) were computed for IDIFs from 6 cylindrical LV VOIs ranging in volume between 0.2 to 35 mL, which were created by eroding and dilating the original 6.5 mL VOI. Between the largest and the smallest VOIs, the estimated β_{AUC} differed by on average 0.2%±5.5% , demonstrating that this correction is not highly sensitive to VOI size. VOI size had slightly greater impact on β_s and β values estimated by the PVC method, with 3.7%±4.4% and 7.4%±8.1% spreads, respectively, between the correction factors computed from the smallest and largest VOIs, with the smallest VOI having the highest recovery.

Fig. S3 Comparison of image-derived input function correction model fits via F-tests. Each point represents one acquisition for one subject. Green line marks critical value for the F-statistic. A. Comparing 1-parameter PVC model to 2-parameter PVC model. B. Comparing scaling to 2-parameter PVC model. PVC = partial volume correction

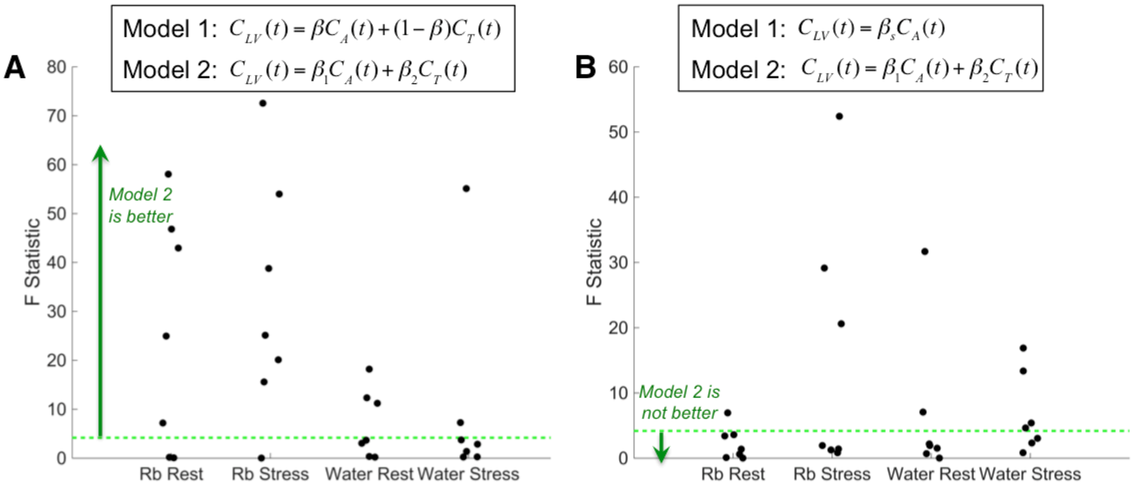

Fig. S4 Comparison of parametric images generated with a 3-parameter fit (without the right ventricle spillover correction term, V_{RV}) and with a 4-parameter fit (with V_{RV}) for one subject (same subject as in Figure 3). All images were generated using the scaled image derived input functions. Including the right ventricle spillover term, V_{RV}, in the kinetic model has little effect on voxels, except for the septal region that is directly adjacent to the right ventricle blood pool (A) K_1 parametric images. The septum appears slightly thinner in the K_1 images when the V_{RV} term is not included. (B) k_2 parametric images. The k_2 images are less affected by the V_{RV} term for ^{15}O-water, and virtually unaffected for ^{82}Rb. (C) V_A and V_{RV} images. When the V_{RV} term is not included, right ventricle spillover is compensated by the V_A term, i.e., the V_A image in the 3-parameter model is very similar to the sum of the V_A and V_{RV} images from the 4-parameter model

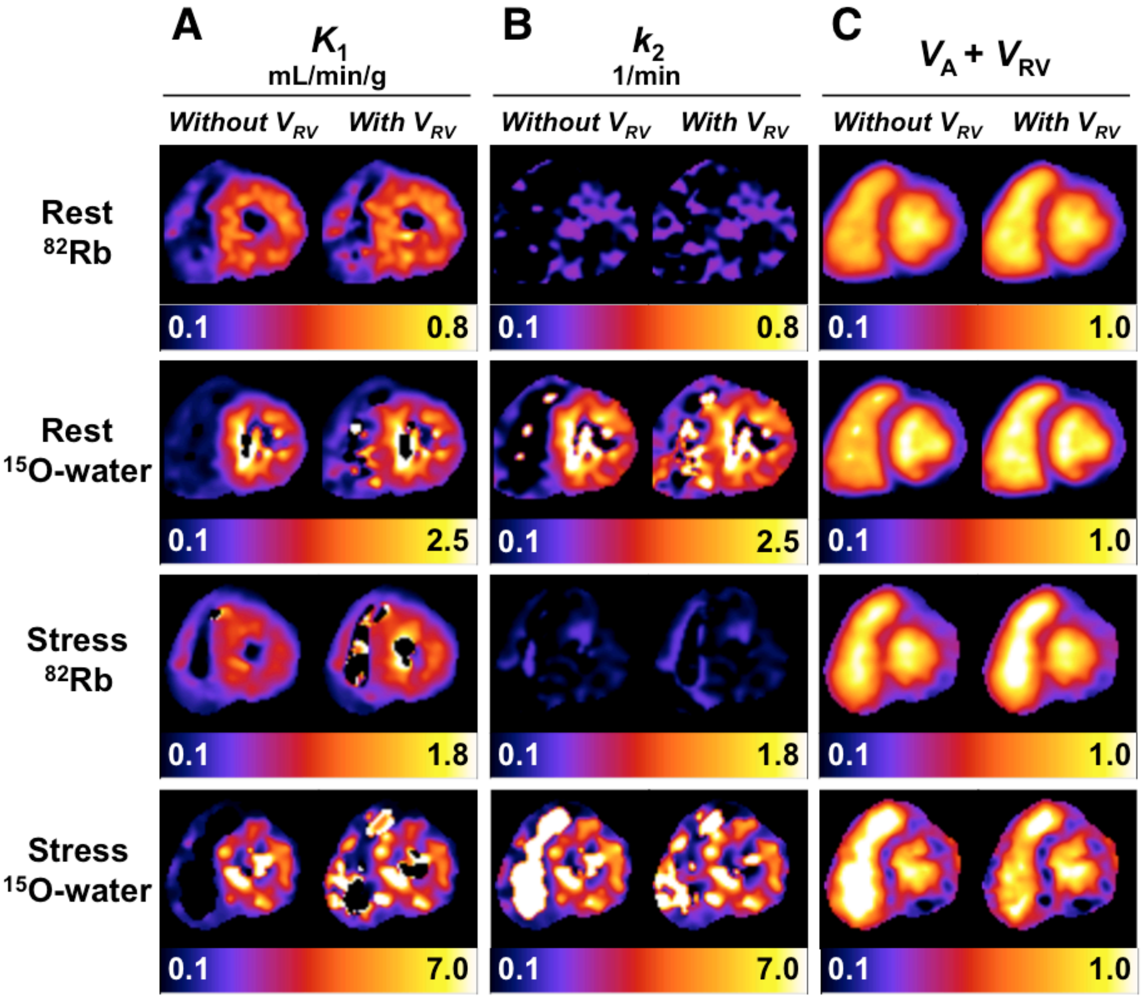

Fig. S5 Example VOIs used for subregional analysis, overlaid on a K_1 image. Blue voxels represent the septal VOI; red voxels represent the lateral wall VOI. VOI=volume of interest. HLA = horizontal long axis. SA = short axis

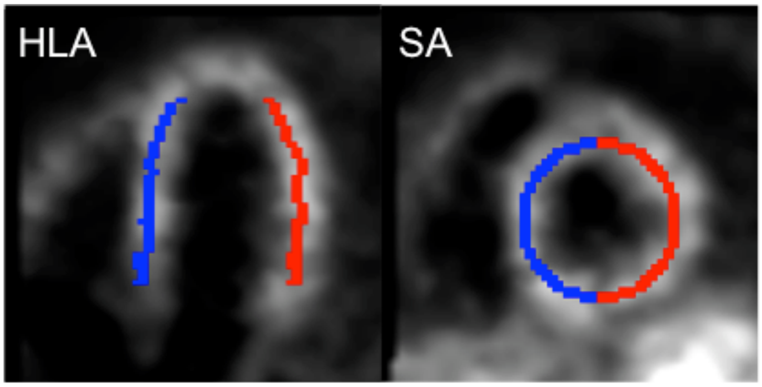

Table S2 Comparison of mean kinetic parameters, estimated with 3- and 54-parameter models, i.e., with and without the right ventricle spillover term (V_{RV}), for whole myocardium (W), lateral wall (L), and septum (S). Omitting the V_{RV} term does not have a substantial impact on regional mean K_1 or k_2 estimates from the whole myocardium (note the very small values of V_{RV}), though the mean septal K_1 values are greater when including the V_{RV} term. Even with the 4-parameter model, there are slight differences between septal and lateral values, in part because the model does not include partial volume mixing with the lung (which has greater impact on the lateral wall), or the liver. V_A estimates were always lower in the septum when V_{RV} was included. Non-negativity constraints were not placed on V_{RV}, and voxels in the lateral wall were prone to having negative V_{RV} estimates

			Uncorrected IDIF		Scaled IDIF	
			**Without V_{RV}**	**With V_{RV}**	**Without V_{RV}**	**With V_{RV}**
**K_1 mL/min/g mean±SD**	**^{82}Rb Rest**	***W***	0.53±0.06	0.55±0.07	0.45±0.05	0.47±0.05
		L	0.52±0.06	0.53±0.07	0.45±0.05	0.46±0.05
		S	0.54±0.07	0.56±0.07	0.45±0.06	0.47±0.06
	**$H_2{}^{15}O$ Rest**	***W***	0.91±0.16	0.95±0.16	0.86±0.15	0.94±0.21
		L	0.91±0.15	0.92±0.15	0.87±0.14	0.88±0.14
		S	0.91±0.18	0.99±0.19	0.86±0.17	1.00±0.31
	**^{82}Rb Stress**	***W***	1.30±0.17	1.41±0.18	1.11±0.13	1.19±0.13
		L	1.26±0.14	1.40±0.16	1.08±0.11	1.18±0.12
		S	1.35±0.21	1.43±0.22	1.14±0.16	1.20±0.16
	**$H_2{}^{15}O$ Stress**	***W***	3.68±0.89	3.94±0.96	3.53±0.85	3.78±0.92
		L	3.77±1.01	3.83±0.98	3.62±0.97	3.67±0.93
		S	3.59±0.83	4.05±1.03	3.44±0.79	3.89±0.99
**k_2 1/min mean±SD**	**^{82}Rb Rest**	***W***	0.13±0.04	0.13±0.04	0.13±0.04	0.13±0.04
		L	0.14±0.04	0.14±0.04	0.14±0.04	0.14±0.04
		S	0.11±0.03	0.12±0.03	0.11±0.03	0.12±0.03
	**$H_2{}^{15}O$ Rest**	***W***	1.05±0.22	1.09±0.21	1.05±0.22	1.09±0.21
		L	1.12±0.22	1.11±0.21	1.12±0.22	1.11±0.21
		S	0.99±0.23	1.07±0.23	0.99±0.23	1.07±0.23
	**^{82}Rb Stress**	***W***	0.23±0.08	0.23±0.09	0.23±0.08	0.23±0.09
		L	0.25±0.10	0.24±0.10	0.25±0.10	0.24±0.10
		S	0.21±0.07	0.22±0.08	0.21±0.07	0.22±0.08
	**$H_2{}^{15}O$ Stress**	***W***	4.10±1.06	4.22±1.11	4.10±1.06	4.22±1.11
		L	4.30±1.20	4.20±1.14	4.30±1.20	4.20±1.14
		S	3.89±0.94	4.24±1.10	3.89±0.94	4.24±1.10

table continued on next page

			Uncorrected IDIF		Scaled IDIF	
			**Without V_{RV}**	**With V_{RV}**	**Without V_{RV}**	**With V_{RV}**
V_A	^{82}Rb Rest	W	0.40±0.05	0.38±0.05	0.37±0.04	0.35±0.04
		L	0.36±0.05	0.37±0.04	0.33±0.04	0.34±0.04
		S	0.46±0.05	0.40±0.06	0.42±0.05	0.36±0.05
	$H_2{}^{15}O$ Rest	W	0.34±0.06	0.29±0.06	0.33±0.05	0.29±0.06
		L	0.30±0.05	0.30±0.05	0.29±0.05	0.29±0.05
		S	0.38±0.07	0.29±0.08	0.36±0.07	0.28±0.07
	^{82}Rb Stress	W	0.44±0.06	0.45±0.07	0.40±0.06	0.42±0.06
		L	0.41±0.06	0.46±0.07	0.38±0.06	0.43±0.07
		S	0.46±0.07	0.44±0.08	0.43±0.06	0.41±0.07
	$H_2{}^{15}O$ Stress	W	0.28±0.06	0.24±0.06	0.27±0.06	0.23±0.06
		L	0.26±0.05	0.29±0.06	0.25±0.05	0.28±0.06
		S	0.31±0.08	0.19±0.07	0.30±0.07	0.19±0.07
V_{RV}	^{82}Rb Rest	W	-	0.024±0.018	-	0.024±0.018
		L	-	-0.013±0.008	-	-0.013±0.008
		S	-	0.064±0.030	-	0.064±0.030
	$H_2{}^{15}O$ Rest	W	-	0.040±0.028	-	0.040±0.028
		L	-	0.000±0.015	-	0.000±0.015
		S	-	0.084±0.043	-	0.084±0.043
	^{82}Rb Stress	W	-	-0.016±0.020	-	-0.016±0.020
		L	-	-0.051±0.017	-	-0.051±0.017
		S	-	0.022±0.035	-	0.022±0.035
	$H_2{}^{15}O$ Stress	W	-	0.029±0.018	-	0.029±0.018
		L	-	-0.014±0.016	-	-0.014±0.016
		S	-	0.076±0.025	-	0.076±0.025

SD=standard deviation
AIF = arterial sample-based input function
IDIF = image derived input function
W = whole myocardium region
L = lateral wall sub-region
S = septum sub-region
N/A = not applicable

Fig. S6. Renkin-Crone model fits by region, using scaled image-derived input functions and no right ventricle spillover term. Solid lines are the curves of best fit, and the dashed lines are 95% confidence intervals. Including the right ventricle spillover term (data not shown) causes the results of Renkin-Crone model fits for septal and lateral regions to be virtually identical

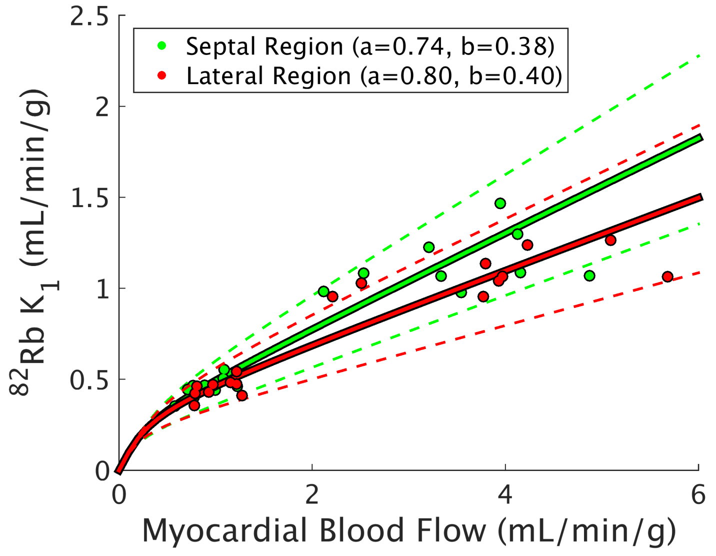

Fig. S7 Comparison of ^{15}O-water MBF as determined from K_1 or k_2. With ^{15}O-water, MBF can be estimated from either the K_1 or k_2 image. Solid line represents fit by Deming regression, accounting for error in both variables. Dashed line represents identity

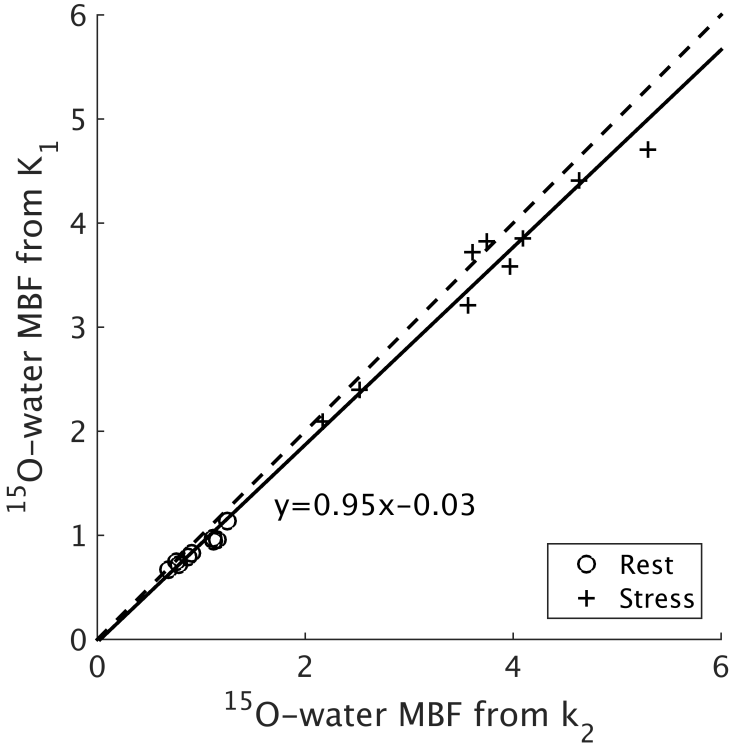

Fig. S8 When using K_1, the partial volume fraction is estimated as 1-V_A, which is not equivalent to the perfusable tissue fraction in all voxels. (a) Voxelwise partial volume fraction 1-V_A is plotted versus perfusable tissue fraction $\alpha = (1-V_A)K_1/(0.91k_2)$ for one ^{15}O-water acquisition. (b) Illustration of voxels included in septal region (green) or lateral region (blue). Voxels from either region with greater than 25% mismatch between partial volume fraction and perfusable tissue fraction are displayed in red.

The K_1-based MBF is expected to be lower than the k_2-based MBF in voxels where the geometric partial volume fraction overestimates the perfusable tissue fraction. This mis-estimation occurs because the term (1-V_A) only accounts for partial volume mixing between the LV and myocardium. Even when the V_{RV} term is included to account for partial volume mixing with the right ventricle, there is still partial volume mixing between the myocardium and lung in the lateral wall that is not accounted for by the geometric partial volume correction

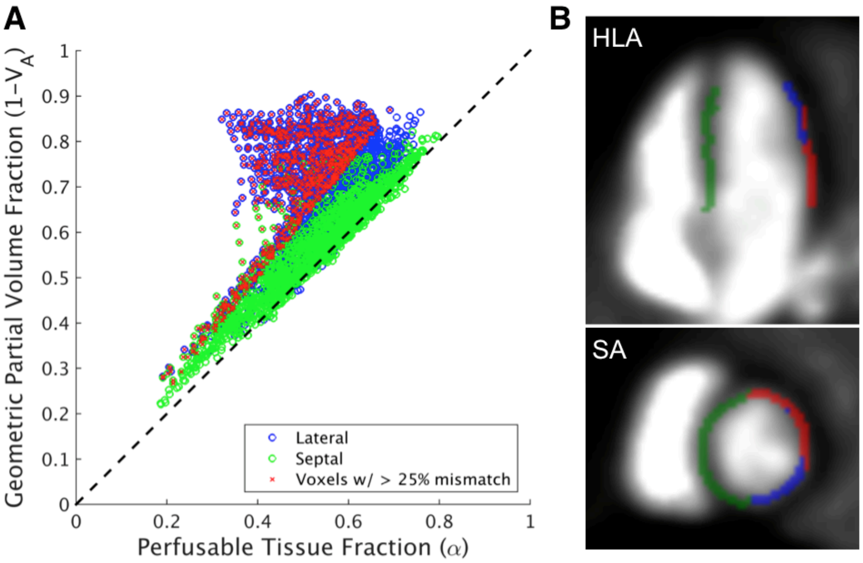

Fig. S9 To demonstrate the statistical improvement in parametric images afforded by TOF and PSF modeling, one subject's data (acquired at rest) were reconstructed with and without TOF or PSF modeling, for comparison. Parametric images were generated for these standard reconstructions, in addition to those from the advanced reconstructions. The theoretical standard error was estimated at each voxel of the parametric images, approximated using the sensitivity matrix (containing the gradient of the kinetic model with respect to the parameters, evaluated at each of the image frame times) and weighted sum-of-squared errors from the model fit. The mean standard errors of ^{82}Rb K_1 and ^{15}O-water k_2 were reduced by 38% and 35%, respectively, when generated from advanced reconstructions, as compared to parametric images generated from standard reconstructions.

The K_1 standard error for ^{82}Rb is higher than k_2 standard error for ^{15}O-water, despite the lower absolute values of K_1, because of partial volume correction: error in V_A propagates into error in K_1. When V_A is close to 1, the computation for K_1 includes division by a small number, resulting in very large K_1. On the other hand, the range of k_2 is inherently limited by the basis function method, so the standard error of k_2 is constrained.

The estimated standard error of parametric images for one subject using conventional vs. advanced reconstruction are shown here in short axis orientation. The background signal outside the heart has been manually masked out. Both acquisitions were at rest. Advanced reconstruction includes time-of-flight and point spread function modeling, whereas standard reconstruction uses neither

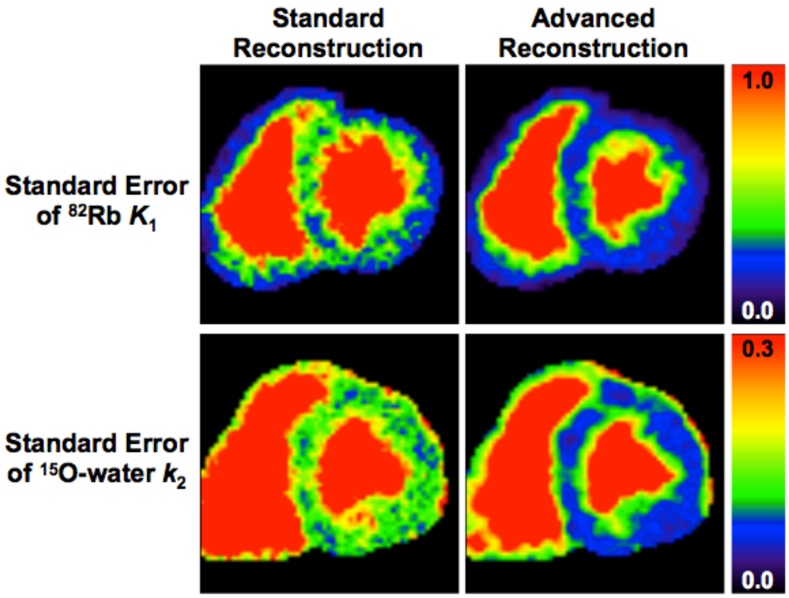

Fig. S10 Comparing unweighted ODR and weighted ODR for fitting the Renkin-Crone model, using scaled IDIFs and no right ventricle spillover term. In this study, weights were set to the reciprocal of the variance of voxel values in the myocardium volumes of interest. Omitting the weighting causes the fit at low flows to fall above the majority of the rest datapoints. Previous studies have not accounted for errors in the explanatory variable, which may have introduced bias into their parameter estimates. ODR = orthogonal distance regression. IDIF = image derived input function. Dashed lines are 95% confidence intervals

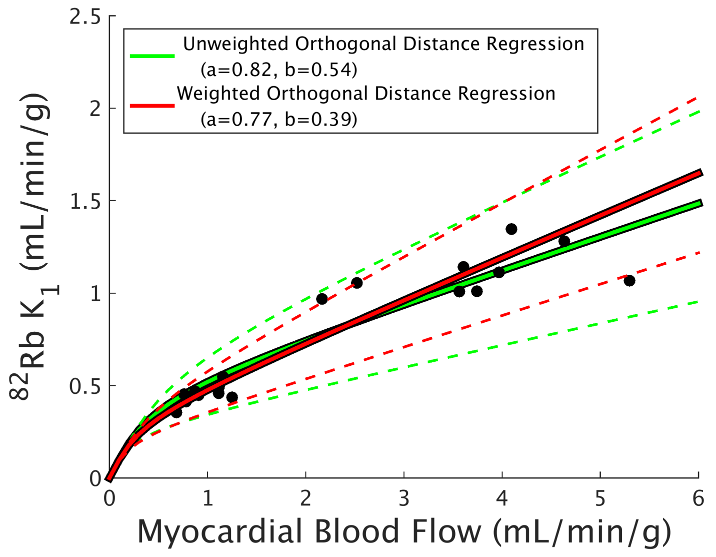

Supplement: Additional file 1: — Figures S1–S10 and Tables S1–S2. (PDF 2.93 mb) [file 13550_2016_215_MOESM1_ESM.pdf]
